# Recent clinical trials with stem cells to slow or reverse normal aging processes

**DOI:** 10.3389/fragi.2023.1148926

**Published:** 2023-04-06

**Authors:** Ricardo P. Garay

**Affiliations:** ^1^ Pharmacology and Therapeutics, Craven, 91360 Villemoisson-sur-Orge, France; ^2^ CNRS, National Centre of Scientific Research, Paris, France

**Keywords:** aging, clinical trial, frailty, lomecel-B, mesenchymal stem cells, rejuvenation, skin aging, stem cells

## Abstract

Aging is associated with a decline in the regenerative potential of stem cells. In recent years, several clinical trials have been launched in order to evaluate the efficacy of mesenchymal stem cell interventions to slow or reverse normal aging processes (aging conditions). Information concerning those clinical trials was extracted from national and international databases (United States, EU, China, Japan, and World Health Organization). Mesenchymal stem cell preparations were in development for two main aging conditions: physical frailty and facial skin aging. With regard to physical frailty, positive results have been obtained in phase II studies with intravenous Lomecel-B (an allogeneic bone marrow stem cell preparation), and a phase I/II study with an allogeneic preparation of umbilical cord-derived stem cells was recently completed. With regard to facial skin aging, positive results have been obtained with an autologous preparation of adipose-derived stem cells. A further sixteen clinical trials for physical frailty and facial skin aging are currently underway. Reducing physical frailty with intravenous mesenchymal stem cell administration can increase healthy life expectancy and decrease costs to the public health system. However, intravenous administration runs the risk of entrapment of the stem cells in the lungs (and could raise safety concerns). In addition to aesthetic purposes, clinical research on facial skin aging allows direct evaluation of tissue regeneration using sophisticated and precise methods. Therefore, research on both conditions is complementary, which facilitates a global vision.

## 1 Introduction

Stem cells (SCs) are undifferentiated cells which can proliferate indefinitely or differentiate into progenitor cells and end-phase differentiated cells (becoming pluripotent) (Mayo, 2021; Slack, 2022). Human embryonic SCs (*h*E-SCs) are found in the inner cell mass of the blastocyst; *h*E-SC research raises ethical concerns (Lo and Parham, 2009), and *h*E-SC transplantation *in vivo* can lead to the formation of large tumors called teratomas (Blum and Benvenisty, 2008).

Small numbers of adult SCs are found in some organ “niches”, including the bone marrow, where hematopoietic progenitor cells (HPC) replenish blood and immune cells. In 1958, Mathe et al. (1959) successfully performed the first adult SC therapy on five workers who had received high-dose accidental irradiation at the Vinca Nuclear Institute in Yugoslavia. After transfusions and grafts of homologous adult bone marrow, all workers survived (Mathe et al., 1959).

For years, the human umbilical cord was a waste material and, unlike *h*E-SCs, its use does not raise ethical concerns. In 1988, Gluckman et al. (1989) successfully performed the first human cord blood transplant in a child with Fanconi’s anemia. Since then, numerous public and private cord blood banks have been established worldwide for the cryopreservation of cord blood in view of its transplantation (Gluckman, 2011).

**FIGURE 1:**
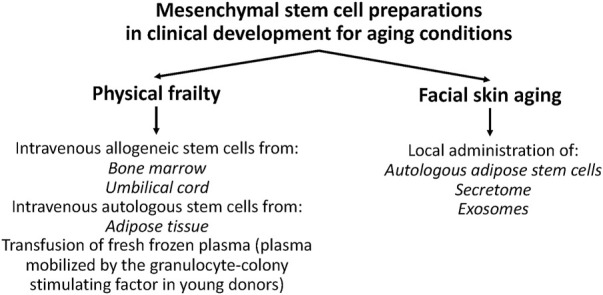
Mesenchymal stem cell (MSC) preparations in clinical development for aging conditions. MSC preparations are in development for physical frailty in older persons and facial skin aging. Reducing aging frailty can increase healthy life expectancy and decrease costs to the public health system. Clinical trials for facial skin aging are important because tissue regeneration is *directly assessed* by sophisticated and precise methods, and because cell entrapment in the lungs and safety issues of intravenous stem cells are avoided.

In the United States, the only SC products that are approved by the FDA consist of allogeneic HPC from human cord blood, for use in patients with disorders affecting the hematopoietic system (FDA, 2020) (Figure 1). Such HPC cord blood products include: Allocord (SSM Cardinal Glennon Children’s Medical Center), Clevecord (Cleveland Cord Blood Center), Ducord (Duke University School of Medicine), Hemacord (BHI Therapeutic Sciences), and some other HPC cord blood preparations (FDA, 2022).

In the EU, the EMA has approved two SC products for disorders that do not affect the hematopoietic system: 1) darvadstrocel (Alofisel^®^, Takeda Ireland), an allogeneic adipose-derived SC preparation to treat perianal fistulas in adults with Crohn’s disease, and 2) holoclar (Holoclar^®^, Holostem Terapie Avanzate, Italy), an autologous corneal SC preparation for severe corneal SC deficiency caused by burns (Cynober, 2020). Several other SC products have been approved in South Korea, Japan, India, Canada and New Zealand (Levy et al., 2020).

Adult mesenchymal stem cells (MSCs) have been extensively investigated in clinical trials (Squillaro et al., 2016). In particular, human bone marrow MSCs (*h*BM-MSCs) have been widely used for clinical research, although they are obtained with low yields, through an invasive procedure (BM aspiration) (Varghese and Mosahebi, 2017) and their ability to proliferate and differentiate declines with age (Rao and Mattson, 2001).

In 2006, Lu et al., (2006) published a protocol to isolate abundant MSCs by enzymatic digestion of the human umbilical cord (hUC) and cell culture expansion. The UC is an easily accessible fetal tissue, and the *h*UC, which was previously discarded as waste material, quickly became an alternative source of MSCs to be investigated in clinical trials (Figure 1).

Another source of human SCs is adipose tissue (*h*AD-MSCs) (Coleman, 1994; Varghese and Mosahebi, 2017; Alexander, 2019; Khazaei et al., 2021; Surowiecka and Struzyna, 2022). Subcutaneous fat tissue contains many more SCs than bone marrow, large amounts of autologous *h*AD-MSCs are easily obtained by liposuction, and autologous *h*AD-MSCs do not require cell expansion.

Aging is associated with a decline in the regenerative potential of adult SCs, and this may play a crucial role in the pathogenesis of age-associated conditions (Rao and Mattson, 2001; Choudhery et al., 2014; Verdijk et al., 2014; Picerno et al., 2021; Zhu et al., 2021). Indeed, the use of SC preparations for aging conditions has a strong rationale:

1. Animal and human studies have shown that as they age, SCs decrease in number and tend to lose their potential for self-renewal and tissue-regeneration [for recent reviews, see (Picerno et al., 2021; Zhu et al., 2021)]. In human skeletal muscle biopsies, Verdijk et al. (2014) found that atrophy of type II (“fast-twitch”) muscle fibers with aging is accompanied by a specific decrease in SC (“satellite cell”) content.Choudhery et al. (2014) showed that *h*AD-MSCs from individuals older than 60 years displayed senescent characteristics compared to cells isolated from young donors, concomitant with reduced viability, proliferation, and differentiation potential.
2. Animal studies showed increased life expectancy with MSC transplantation. In mice, Shen et al. (2011) reported that transplantation of young MSCs prolongs the life span of old mice. Mansilla et al. (2016) found that intravenous administration of *h*BM-MSC to a 6-month-old rat increased its lifespan to 44 months, compared to an average of 36 months in control animals. Lavasani et al. (2012) showed that intraperitoneal administration of muscle-derived stem/progenitor cells from young wild-type mice significantly increased the lifespan and healthspan of progeroid mice (a rodent model of accelerated aging).
3. Within the animal kingdom, the healthy life expectancy of different animal species depends to a large extent on the regenerative capacity of their SC, notably in invertebrates such as planarians and hydra (Handberg-Thorsager et al., 2008).

In recent years, several clinical trials have been launched to evaluate the efficacy and safety of SCs on aging conditions (Figure 1). Here, those SC preparations were identified, and trials were analyzed from information extracted from national (United States, EU, China, and Japan) and international (World Health Organization, WHO) clinical trial databases. Stem cell-based therapies for age-related diseases are described elsewhere (Levy et al., 2020; Rezaie et al., 2022).

## 2 Methods

### 2.1 Identification of recent clinical trials with stem cell preparations for aging

#### 2.1.1 Clinical trials databases

Clinical trials databases from the US (ClinicalTrials.gov), EU (clinicaltrialsregister.eu), China (chictr.org.cn/searchprojen.aspx), Japan (https://rctportal.niph.go.jp/en/) and the World Health Organization (WHO; trialsearch.who.int) were accessed to identify recent clinical trials with SC preparations for aging conditions, using a previously developed approach (Garay et al., 2016; Garay, 2021) slightly modified. The WHO Clinical Trials Search Portal provided access to trials registered in 14 primary registries (Australia, Brazil, Cuba, Germany, India, Iran, ISRCTN, Korea, Lebanon, Netherlands, Pan African, Peru, Sri Lanka, and Thailand). For each website, the list of clinical trials was obtained by filling out the “Advanced Search” form (or “More Information” form).

#### 2.1.2 Selection criteria

In order to be retained for this review, compounds needed to be in clinical trials with SC interventions for aging conditions and satisfy the following criteria:

1. Trial declared with “Aging” as “Condition”, “stem cell” as “Other terms”, and “Interventional Studies (Clinical trials)” as “Study type”
2. Trial updated on 1 January 2019 or later,
3. Trial not terminated,
4. Trial including healthy participants.

### 2.2 Data extraction and organization

For each selected clinical trial, the following relevant data were extracted: identifier number (and/or designated name, and/or bibliographic reference), aging condition, SC preparation, trial sponsor(s), main outcomes, duration of the study, number of patients, and trial status (results, if available, or expected completion date). Clinical trials were listed according to the aging condition and SC preparation investigated.

### 2.3 Additional sources of information

Relevant articles related to the selected SC interventions were searched in the following biomedical literature databases: PubMed (https://pubmed.ncbi.nlm.nih.gov), Science Direct (www.sciencedirect.com/search), Cochrane Library (www.cochranelibrary.com), and Google Scholar (https://scholar.google.com). For each website, relevant articles were found by using the name of the SC preparation, OR the clinical trial identifier number AND “aging condition”. Clinical trial information was also obtained by consulting the websites of pharmaceutical and biotechnology companies working in the field of stem cells and aging.

### 2.4 Analysis

The current treatment and therapeutic needs of each aging condition were identified in the corresponding clinical practice guidelines (CPG). The therapeutic impact of the selected clinical trials was evaluated in the context of such competitive environment. Research analysis included four therapeutic aspects: 1) Key findings from SC interventions for aging, 2) research with ongoing clinical trials, 3) clinical trial limitations, and 4) future perspectives.

## 3 Clinical trials with SC preparations for aging

Clinical trial registries were accessed from 1 August 2022 to 16 January 2023, to identify trials with SC preparations for aging. The US database (ClinicalTrials.gov), included twenty-three clinical trials updated on 1 January 2019 or later. Of these, NCT03457870, NCT02642094, NCT04712955, NCT02456870, NCT01169831, NCT02790541, NCT03140319, NCT03535844, NCT04450602, and NCT04450589 were excluded from the present analysis because the interventions did not meet the inclusion criteria. Thirteen clinical trials met the inclusion criteria and were included in the analysis (Tables 1–3).

**TABLE 1:** Recent clinical trials with allogeneic stem cell preparations for physical frailty in older persons (2019 and later).^a^

SC preparation	Sponsor	Identifier	Main outcomes	Time frame	N^c^	Results or status
*h*BM-MSCs^d^	Longeveron (United States)	NCT02065245	Safety^e^	1 month	15	Safe
*h*BM-MSCs^d^	Longeveron (United States)	NCT02065245	Efficacy^f^	6 months	30	Positive
*h*BM-MSCs^d^	Longeveron (United States)	NCT03169231	6 MWD	180 days	150	Completed
*h*BM-MSCs^d^	Longeveron (United States)	NCT02982915	Vaccine adjuvant^h^	12 months	62	September 2021^g^
*h*BM-MSCs^d^	Longeveron (Japan)	jRCT2043200038	6 MWD	180 days	45	NC
*h*vBM-MSCs^g^	VA’s ORD (United States)^i^	NCT05284604	Adherence^j^	6 months	36	June 2025^g^
*h*UC-MSCs	Shanghai East Hosp (CHN)	NCT04314011	Safety and Efficacy	1 and 6 months	30	Completed
*h*UC-MSCs	Vinmec Research (VNM)^k^	NCT04919135	Safety and Efficacy	12 months	44	Not yet recruiting
*h*UC-MSCs	FOREM (United States)	NCT05018767	Safety	4 years	20	November 2025^g^
*h*AD-MSCs^l^	Healeon Medical (United States)	NCT03514537	Safety (Frailty)	6 months	200	March 2023^g^
GMFFP^m^	Maharaj Institute (United States)	NCT03458429	Safety (Efficacy^n^)	24 M	30	February 2023^g^

^a^ Most of the studies were randomized controlled trials (see text for details).

^b^ Abbreviations: 6 MWD, 6-min walk distance; allo*-h*MSCs, allogeneic mesenchymal stem cells; FOREM, Foundation for Orthopaedics and Regenerative Medicine; *h*BM-MSCs, human bone-marrow mesenchymal SCs. Hosp, hospital. *h*UCM-SCs, human umbilical cord mesenchymal stem cells; NC, not communicated.

^c^ Number of participants.

^d^ Lomecel-B (also called “allo-*h*MSCs”).

^e^ Phase I safety trial, including frailty outcomes.

^f^ Phase II RCT, investigating 1-month safety and 6-month efficacy on aging frailty.

^g^ Primary completion date (past or estimated).

^h^ Phase II RCT, to test the efficacy of Lomecel-B to improve influenza vaccine responses (12 months), including an initial phase I safety trial (30 days). Vertebral *h*BM-MSCs.

^i^ Veterans Health Administration-Office of Research and Development.

^j^ Percentage of study visits attended.

^k^ Vinmec Research Institute of Stem Cell and Gene Technology (Vietnam).

^l^ Cellular Stromal Vascular Fraction, an autologous *h*AD-MSCs, preparation.

^m^ GCSG-Mobilized Fresh Frozen Plasma.

^n^ Frailty Index and other secondary outcomes.

**TABLE 2:** Recent clinical trials with stem cell preparations for facial skin aging and photoaging (2019 and later).^a^

SC preparation	Sponsor	Identifier	Outcomes^b^	Time frame	N^c^	Results or status^b^
SVF^d^	Xuzhou Medical Univ (CHN)	NCT02923219	Volume; skin quality	6 months	50	Positive^e^
SVF^d^	Alexandria Univ (Egypt)	NCT03928444	Facial rejuvenation	6 months	15	Completed
SVF^d^	HA Hospital (Cuba)^f^	RPCEC00000362	Wrinkles and furrows	1 year	N.C.	December 2022^g^
SVF^d^	Tehran Univ (Iran)^h^	IRCT20141007019432N2	Wrinkles	6 months	46	Started
SC secretome^i^	SN Yusharyahya^j^	NCT05508191	Facial rejuvenation^k^	6 weeks	30	October 2022^g^
*h*BM-MSCs	Stemedica (United States)^l^	NCT01771679	Safety (Photoaging)	1 year	29	Suspended^m^
SC Exosomes	Sun Yat-sen Univ (China)^n^	ChiCTR2200061216	Photoaging^o^	N.A.	10	December 2024^g^

^a^ Most of the studies were randomized controlled trials (see text for details). N.A., not applicable; N.C, not communicated; SC, stem cell; Univ, University.

^b^ See text for details.

^c^ Number of participants.

^d^ Autologous stromal vascular fraction (SVF).

^e^ The results of the study have been reported by Yin et al. (Yin et al., 2020).

^f^ Hermanos Ameijeiras Surgical Clinical Hospital (Havana).

^g^ Primary completion date (past or estimated).

^h^ Tehran University Medical Sciences and Sinacell Corporation (Tehran).

^i^ *h*AD-MSC, secretome developed by PT, Kimia Farma Tbk (Jakarta, Indonesia).

^j^ Shannaz Nadia Yusharyahya (Indonesia University).

^k^ Skin aging changes evaluated by several methods (see text).

^l^ Stemedica Cell Technologies.

^m^ The study has stopped early but it can start again.

^n^ The seventh Affiliated Hospital of Sun Yat-sen University (Shenzhen).

^o^ *h*AD-MSC, derived exosomes loaded with circcol elns (a circular RNA, circRNA) are tested for their ability to promote collagen and elastin synthesis in skin samples from 6 to 10 photoaged patients (55–75 years).

**TABLE 3:** Other clinical trials with stem cell preaparations for aging (2019 and later).

Intervention	Sponsor	Identifier	Outcomes^a^	Time frame	N^b^	Results or status
NT-020	North Texas Univ (United States)^c^	NCT01847027	Blood SC levels^d^	4 weeks	23	Negative
*h*UC-MSCs and *h*AD-MSCs	Landmark (Malaysia)^e^	NCT04174898	Safety^f^	1 year	100	April 2021^g^

SC, stem cell. Univ, University.

^a^ See text for details.

^b^ Number of participants.

^c^ University of North Texas Health Science Center.

^d^ CD34+ and CD133+ blood cell levels.

^e^ Landmark Medical Centre Sdn Bhd.

^f^ Safety, quality of life and inflammatory markers.

^g^ Primary completion date (past or estimated).

No other clinical trials were found in the EU database (clinicaltrialsregister.eu). The Chinese database (chictr.org.cn/searchprojen.aspx), included one clinical trial (ChiCTR2200061216) (Table 2). The Japanese database (https://rctportal.niph.go.jp/en/) included one clinical trial (jRCT2043200038) (Table 1). The WHO database (trialsearch.who.int) included two clinical trials (IRCT20141007019432N2, and RPCEC00000362) (Table 2).

### 3.1 SC preparations for the frailty of aging

Eleven clinical trials selected for analysis were investigating SC preparations for aging frailty (Table 1). These included six trials with allogeneic human bone marrow MSCs (*h*BM-MSCs), three trials with allogeneic human umbilical cord MSCs (*h*UC-MSCs), one trial with autologous adipose-derived MSCs (*h*AD-MSCs), and one trial with plasma mobilized by the granulocyte-colony stimulating factor (GMFFP).

**FIGURE 2:**
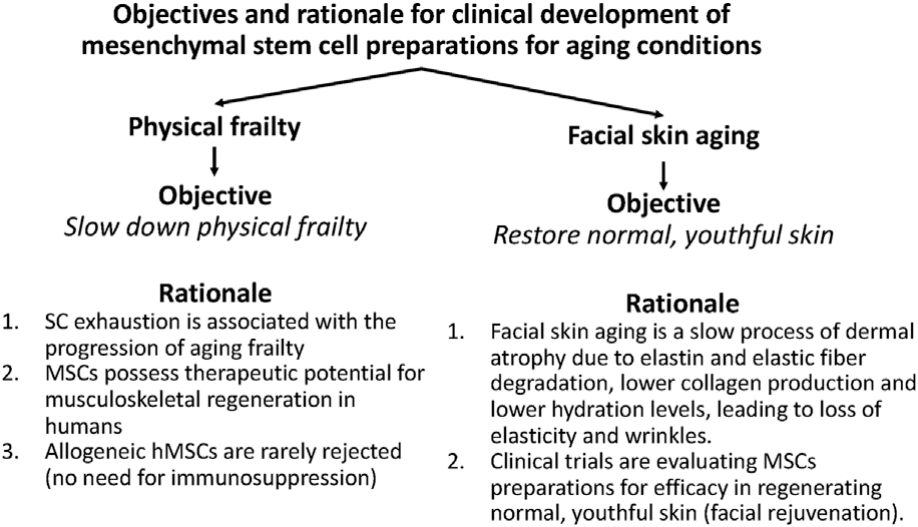
Objectives and rationale for clinical development of mesenchymal stem cell preparations for physical frailty and facial skin aging.

The clinical development of MSCs preparations for physical frailty in older persons has a strong rationale (Figure 2). A large amount of evidence suggests that SC exhaustion is associated with the progression of aging frailty (Verdijk et al., 2014; Sousa-Victor and Munoz-Canoves, 2016; Zhu et al., 2021). In addition, human studies showed that MSCs possess therapeutic potential for musculoskeletal regeneration [for a review, see (Steinert et al., 2012)]. Finally, allogeneic *h*MSCs are rarely rejected, making them suitable for MSC therapy without the need for immunosuppression (Hare et al., 2009; Florea et al., 2019). These observations suggested that an intravenous infusion of allogeneic *h*MSCs may be a potentially effective therapy for physical frailty in older persons (Figure 2).

#### 3.1.1 hBM-MSCs

Lomecel-B (or “allo-*h*MSCs”, Longeveron, United States) is a formulation of allogeneic *h*BM-MSCs sourced from the posterior iliac crest of healthy young adult donors (aged 18–45 years) and expanded in culture (Golpanian et al., 2016; Yousefi et al., 2022). After a specific number of expansion cycles, the cells are harvested, separated into specific doses, and frozen until future use. Unlike an autologous bone marrow transplant (that is used for a single patient), tissue from a single donor is used to obtain many doses of Lomecel-B for use in multiple patients.

Longeveron has launched a clinical development program with intravenous Lomecel-B for aging frailty. The program includes five clinical trials designed to determine if Lomecel-B can improve physical function, reduce inflammation and improve quality of life in frail older adults (Table 1).

##### 3.1.1.1 NCT02065245

CRATUS (NCT02065245) consisted of a phase I open label, escalated dose pilot trial (Golpanian et al., 2017), and a phase II randomized controlled trial (RCT) versus placebo (Tompkins et al., 2017) (Table 1). The phase I open label trial (Golpanian et al., 2017) included 15 elderly subjects (mean age: 78.4 years) with early signs and symptoms of frailty, and a frailty score between 4 and 7 on the Clinical Frailty Scale (CFS) (Rockwood et al., 2005). Participants were divided in three groups (*n* = 5/group) scheduled to receive 20-, 100- or 200-million *h*BM*-*MSCs, delivered *via* peripheral intravenous infusion (Golpanian et al., 2016). No therapy-related TE-SAE (treatment emergent-serious adverse event) occurred during the trial (Golpanian et al., 2017). There were no signs of T-cell activation (a marker of graft rejection) at 6-month. Only one subject (20-million group) developed mild to moderate donor-specific antibodies.

Significant increases in the 6-min walk distance (6 MWD) test were obtained: 1) in the group of 20-million *h*BM*-*MSCs at 6 months (mean value of the increase = 37.2 m), and 2) in the group of 100-million *h*BM*-*MSCs at 3 months (36.6 m) and at 6 months (76.6 m) (Golpanian et al., 2017). No significant increases were seen in the group of 200-million *h*BM*-*MSCs (Golpanian et al., 2017).

TNF-alpha levels (an inflammatory marker) significantly decreased in the groups of 100-and 200-million *h*BM*-*MSCs at 6-month. No significant changes were seen in Interleukin-6 (IL-6) or C-reactive protein (CRP) (Golpanian et al., 2017).

The phase II RCT (Tompkins et al., 2017) included 30 elderly subjects (mean age = 75.5 years) with frailty scores between 4 and 7 on the CFS (Rockwood et al., 2005). Subjects receiving 100 million cells (*n* = 10) or 200 million cells (*n* = 10) were compared with those receiving placebo (*n* = 10). The results confirmed those obtained in the phase I open label trial (Golpanian et al., 2017). In particular, the 6 MWD significantly increased in the 100 M-group from baseline (mean value = 345.9 m) to 6-month (410.5 m). Immuno-tolerability was acceptable (only three participants showed a mild to moderate increase in donor specific antibodies).

##### 3.1.1.2 NCT03169231

The CRATUS trial was limited by its small sample size (Golpanian et al., 2017; Tompkins et al., 2017). NCT03169231 is a phase IIb multicenter RCT evaluating Lomecel-B *versus* placebo (Yousefi et al., 2022) (Table 1). A total of 150 older adults with CFS scores of 5“mildly frail” or 6“moderately frail” (Rockwood et al., 2005), and 6 MWD of >200 m and <400 m was included in the study. Primary outcome is the change from baseline in 6 MWD compared to placebo. Secondary outcomes are changes in overall physical function and TNF-alpha. This trial was recently completed.

In September 2021, Longeveron announced preliminary biomarker results from the NCT03169231 trial (Longeveron, 2021b). Administration of Lomecel-B was accompanied by a statistically significant reduction in serum soluble TIE-2 (sTIE-2) levels, in a dose-dependent manner, compared to placebo. TIE-2 is a cell surface receptor tyrosine kinase that plays a pivotal role in vascular barrier maintenance, and increased levels of sTIE-2 in the blood stream may indicate endothelial dysfunction (Idowu et al., 2020).

##### 3.1.1.3 NCT02982915

HERA (NCT02982915) is a phase I/II RCT to test the safety and efficacy of intravenous Lomecel-B to improve influenza vaccine (fluzone) responses in subjects with aging frailty (Table 1). Following an initial phase I safety trial (30 days), a phase II RCT will assess whether Lomecel-B may be an effective vaccine adjuvant to enhance influenza virus inactivation (assessed by hemagglutination inhibition assays) (time frame: 12 months). Primary completion date was expected for September 2021.

##### 3.1.1.4 jRCT2043200038

jRCT2043200038 is a phase II RCT evaluating intravenous Lomecel-B in Japan (Table 1). The study includes people 70–85 years of age, who present the CHS (Cardiovascular Health Study) frailty phenotype (Fried et al., 2001) and serum levels of TNF-alpha <2.5 pg/mL. Participants are divided into three groups that receive a single intravenous infusion of 50 million *h*BM-MSCs or 100 million *h*BM-MSCs or placebo. The primary outcome is the change in the 6 MWD from baseline to 180 days post-infusion in the high-dose group compared to placebo. Secondary outcomes are: 1) the change in 6 MWD in the low dose group compared to placebo, and 2) the change in TNF-alpha levels in the high dose group. Recruitment status is pending and completion date was not communicated to https://rctportal.niph.go.jp/en/.

#### 3.1.2 hvBM-MSCs

##### 3.1.2.1 NCT05284604

NCT05284604 is a phase I/II RCT investigating *h*BM-MSCs derived from vertebrae (*h*vBM-MSCs, obtained from the vertebral bodies of deceased organ donors) versus placebo (Table 1). *h*vBM-MSCs are administered intravenously to older adults (65–85 years of age) who meet the following conditions: 1) Modified Physical Performance Test (mPPT) score of 18–31, 2) Clinical Frailty Scale (CFS) score of 5 or 6 and, 3) 6 MWD of >200 m and <400 m. The primary outcome is adherence (percentage of study visits attended). Secondary outcomes include: number of participants recruited, mPPT score, CFS score, and 6-min walk test (6 MWT). Other secondary outcomes include: adverse events, inflammatory markers and quality of life. The trial is not recruiting yet. Primary completion date is expected for June 2025.

#### 3.1.3 hUC-MSCs

##### 3.1.3.1 NCT04314011

NCT04314011 is a phase I/II RCT evaluating the safety and efficacy of intravenous *h*UC-MSCs in older adults (60–80 years of age) with a frailty score of 1–4 on the Fried Phenotype Scale (Op het Veld et al., 2015) (Table 1). Participants receive two intravenous infusions of *h*UC-MSCs (10^6^ cells/kg) or saline separated by an interval of 1 month, and are followed for 6 months (after the first intervention). The primary outcome is the occurrence of serious adverse events (SAEs) during the month following the infusion. Secondary out outcomes are changes in: 1) Fried phenotype scale scores (Op het Veld et al., 2015), 2) blood proinflammatory cytokines and 3) quality of life, assessed at baseline, 1 month, 3 months and 6 months. The trial was recently completed, but the results have not yet been published on ClinTrials.gov.

##### 3.1.3.2 NCT04919135

NCT04919135 is a phase I/II RCT investigating the safety and efficacy of adjunctive intravenous administration of allogeneic *h*UC-MSCs in patients receiving standard treatment for frailty in Vietnam [Hightamine (Hankook Korus Pharm, Korea), Total calcium (Nugale Pharmaceutical, Canada), and Bioflex (Ausbiomed, Australia)] (Table 1) (Hoang et al., 2022). The intervention group will receive two doses of *h*UC-MSCs (1.5 × 10^6^ cells/kg) separated by a time interval of 3 months. The primary outcome is the occurrence of treatment-dependent SAEs. Secondary outcome measures include the 6 MWD test and CD3^+^ cells. The trial is not yet recruiting.

##### 3.1.3.3 NCT05018767

NCT05018767 is a single-arm, phase I trial designed to assess the long-term safety of a single intravenous infusion of cultured allogeneic *h*UC-MSCs (100 million cells) in subjects with aging frailty (Table 1). Patients will be evaluated at baseline and at 1, 6, 12, 24, 36, and 48 months. NCT05018767 is currently recruiting participants and primary completion is expected in November 2025.

#### 3.1.4 hAD-MSCs

##### 3.1.4.1 NCT03514537

NCT03514537 is an open trial to investigate the safety (and efficacy) of an autologous preparation of *h*AD-MSCs (cellular SVF, cSVF) for aging frailty (Table 1). The study includes adult and older adults (40–90 years) who have noted compromise to activities or work requirements due to increasing age and loss of energy. Participants receive intravenous infusions of cSVF isolated from subdermal adipose tissue removed from the trunk or upper thigh area. The primary outcome is the occurrence of Treatment-Emergent Adverse Events (TEAEs) during 6 months following the infusion. Secondary outcomes are changes in weight, activity level, mobility, and fatigue (at 6 months). NCT03514537 is currently recruiting participants and primary completion is expected in March 2023.

#### 3.1.5 GMFFP

Granulocyte-colony stimulating factor (G-CSF) stimulates the BM to produce granulocytes and SCs, and release them into the bloodstream (Patterson and Pelus, 2017). GMFFP (GCSF-Mobilized Fresh Frozen Plasma) is a fresh frozen plasma preparation harvested from young, healthy donors (Maharaj, 2020) (Table 1).

##### 3.1.5.1 NCT03458429

NCT03458429 is a single-arm, phase I/II trial of GMFFP in elderly (55–95 years) and frail people (score of 4–7 on the Clinical Frailty Scale and/or abnormal Immune Risk Profile) (Table 1). Participants receive 12 once monthly transfusions of GMFFP (initial treatment period of 12 months) and are followed for a total of 24 months. The primary outcome is the number of participants with treatment-related adverse events. Secondary efficacy outcomes include frailty index (mobility, energy, strength, physical activity, nutritional status, mood, cognition, and social support), immune risk profile, and cognitive function. Primary completion date was expected for February 2023.

### 3.2 SC preparations for facial skin aging and photoaging

Skin aging is due to natural causes, as well as extrinsic factors (especially Sun exposure: Photoaging) (Zhang and Duan, 2018; Wong and Chew, 2021). Several SC preparations are investigated for facial skin aging and photoaging (Table 2).

Long-term natural aging is a slow process of dermal atrophy due to elastin and elastic fiber degradation, lower collagen production and lower hydration levels, leading to loss of elasticity and wrinkles (Zhang and Duan, 2018; Wong and Chew, 2021). Clinical trials with MSCs preparations for facial skin aging are evaluating their efficacy in regenerating normal, youthful skin (facial rejuvenation) (Figure 2).

Exposure to ultraviolet (UV) radiation facilitates skin aging (photoaging), characterized by the degradation of collagen and elastin, with deposition of collagen breakdown products and abnormal elastin fibers in the dermis (solar elastosis) (Huang and Chien, 2020). Clinical trials with MSCs preparations are evaluating their efficacy in restoring a normal skin.

#### 3.2.1 Stromal vascular fraction (SVF) for facial skin aging

The “stromal vascular fraction” (SVF) is a preparation of autologous *h*AD-MSCs obtained by liposuction, followed by collagenase digestion, filtration, centrifugation and separation of the SVF (Coleman, 1994; Varghese and Mosahebi, 2017; Alexander, 2019; Khazaei et al., 2021; Surowiecka and Struzyna, 2022). The SVF represents about 10% of the adipose tissue volume, and is composed of *h*AD-MSCs, adipocyte progenitors, fibroblasts, endothelial cells, vascular smooth muscle cells, lymphocytes, and a variety of immune cells (T-cells and M2 macrophages). The efficacy and tolerability of SVF-enriched autologous fat grafting is currently being investigated in facial skin aging.

##### 3.2.2.1 NCT02923219

NCT02923219 was an RCT comparing the efficacy of SVF-assisted autologous fat grafting (intervention) versus fat transfer alone (control) for facial volume restoration and improvement of skin quality (Table 2) (Yin et al., 2020). Fifty women (mean age: 35.4 years) participated in the study. At 6 months: 1) Whole face volumes (assessed by 3D scanner and Geomagic software) were significantly higher in the intervention group (77.6%) compared to the control group (56.2%, *p* < 0.001), 2) wrinkles and texture (assessed by VISIA detector) improved significantly more in the intervention group than in the control group, and 3) graft survival rate was significantly higher in the intervention group than in the control group.

##### 3.2.2.2 NCT03928444

NCT03928444 is an RCT comparing intradermal autologous SVF injection on one side of the face versus saline injection on the other side (Table 2). Fifteen female participants with facial aging (35 years or older) are included in the study and will be followed for 6 months. The primary outcome is the degree of aesthetic improvement using the global aesthetic improvement GAIS 5-point scale (Savoia et al., 2014). The trial was completed, but the results were not posted to ClinicalTrials.gov.

##### 3.2.2.3 RPCEC00000362

RPCEC00000362 is an RCT (single-blind) comparing the efficacy of SVF-enriched fat transfer versus conventional fat transfer (Table 2). Participants with facial aging (30–59 years of age) are included in the study and will be followed for 12 months. Outcomes include clinical evaluation and evolution of furrows and wrinkles. Trial completion date was expected for December 2022.

##### 3.2.2.4 IRCT20141007019432N2

IRCT20141007019432N2 is a single-arm clinical trial, designed to investigate the efficacy of autologous SVF transplantation in reducing facial wrinkles (Table 2). Forty-six (46) participants with facial aging (35–65 years of age and with grade 2 to 4 wrinkle type) were included in the study and will be followed for 6 months. The primary outcome is biometric evaluation (with visioface and skin ultrasound) of the amount and extent of facial wrinkles. The trial completion date was not reported.

#### 3.2.2 Soluble paracrine SC factors (secretome) for facial skin aging

##### 3.2.2.1 NCT05508191

NCT05508191 is a single-blind RCT comparing two methods of AD-MSCs “secretome” administration for facial aging (fractional CO₂ laser treatment on one side of the face and microneedle treatment on the other half) (Table 2; Figure 1). The term “secretome” designates the soluble paracrine factors produced by SCs (Xia et al., 2019). Thirty female participants with facial aging (35–59 years) are included in the study and will be followed for 6 weeks. Primary outcomes are: 1) Skin aging changes evaluated by dermoscopy photoaging scale and by Janus-3Ⓡ skin analyzer, 2) skin capacitance evaluated by the CorneometerⓇ and 3) total water content in the stratum corneum of the skin. Primary completion date was expected for October 2022.

#### 3.2.3 Facial photoaging

##### 3.2.3.1 hBM-MSCs

NCT01771679 is a phase I/II safety trial to evaluate the safety and efficacy of a single intravenous injection of allogeneic (non-hematopoietic) *h*BM-MSCs for the treatment of facial photoaging in men and women 40–70 years of age (Table 2). Recruitment was suspended.

##### 3.2.3.2 Exosomes

SCs secrete exosomes (40–120 nm extracellular vesicles), which contain cytokines, growth factors, messenger RNAs, and different non-coding RNAs, especially micro-RNAs (mi-RNAs) (Hamdan et al., 2021) (Figure 1).

###### 3.2.3.2.1 ChiCTR2200061216

ChiCTR2200061216 investigates if *h*AD-MSC derived exosomes loaded with circcol elns (a circular RNA, circRNA) can promote collagen and elastin synthesis in skin samples from 6 to 10 photoaged patients (55–75 years). The study compares samples of facial skin tissue (part exposed to light), with skin tissue of the hip or upper arm (part protected from light) (Table 2). The study is open for recruitment. Completion is expected for December 2024.

### 3.3 Other SC therapies for aging

#### 3.3.1 NT-020

NT-020 (NutraStem^®^) is a patented nutraceutical formulation containing green tea extract, blueberry extract, carnosine, and vitamin D3 (EurekAlert, 2008). In 2006, Bickford et al. (2006) reported that these agents (as well as catechin) synergistically stimulated the *in vitro* proliferation of human bone marrow and human CD34^+^ and CD133+ cells. CD34^+^ are often used clinically to quantify H-SC numbers in H-SC transplantation (Remberger et al., 2020). CD133 is a well-characterized biomarker of normal and cancer SCs (Barzegar Behrooz et al., 2019).

##### 3.3.1.1 NCT01847027

NCT01847027 is a phase II RCT that investigated whether NT-020 in combination with an exercise stimulus was able to increase blood levels of CD34^+^ and CD133+ SCs in persons aged 50–70 years (Table 3). No significant increases in CD34^+^ and CD133+ SCs (primary outcome) were found at 2 or 4 weeks after starting the intervention.

#### 3.3.2 hUC-MSCs and hAD-MSCs for quality of life and morbimortality risk

##### 3.3.2.1 NCT04174898

NCT04174898 is a single-arm, phase I trial investigating safety, quality of life and morbimortality risk of *h*UC-MSC and *h*AD-MSC infusion in adults and older adults (Table 3). Morbimortality risk is assessed by measuring inflammatory markers of aging (IL-6, CRP and TNF-alpha) (Giovannini et al., 2011; Puzianowska-Kuznicka et al., 2016; St Sauver et al., 2022). Primary completion was expected in April 2021. The study is not yet recruiting.

## 4 Discussion

Clinical research with SC interventions for aging has only recently begun. SC interventions are in development for the treatment of two important aging conditions: physical frailty and facial skin aging.

### 4.1 Physical frailty in older persons

Physical frailty in older adults is characterized by reduced locomotor activity and decreased immunological functioning (Fedarko, 2011; Dent et al., 2019). Aging frailty was recognized as a disease in the WHO ICD-11 (International Classification of Diseases, 11th Revision: MG2A ageing associated decline in intrinsic capacity; https://icd.who.int/browse11/l-m/en#/http://id.who.int/icd/entity/835503193). In addition, the International Conference of Frailty and Sarcopenia Research (ICFSR) edited Clinical Practice Guidelines (CPGs) for the identification and management of physical frailty (Dent et al., 2019) [other CPGs for frailty can be found in (Zheng et al., 2022)].

In Western countries, the prevalence of physical frailty is around 15% in adults ≥65 years and increases to more than 25% in adults >85 years (Dent et al., 2019). Locomotion frailty increases the risk of falls, disability and hospitalization (Xue, 2011). The SHARE study (24,634 European people over 50 years of age, followed for 11 years) showed that frailty status was associated with increased all-cause mortality (Grabovac et al., 2019).

Therapeutic interventions for physical frailty have focused on exercise and nutritional supplementation (Dent et al., 2019; Mohd Suffian et al., 2020; Zheng et al., 2022). The ICFSR-CPGs (Dent et al., 2019) recommend a multi-component physical activity programme as first-line therapy for physical frailty in older adults, and protein/caloric supplementation when there is weight loss or malnutrition. There are no specific medical or biological treatments to prevent, delay, or reverse aging frailty (Tompkins et al., 2017; Cesari et al., 2022), and the ICFSR-CPGs (Dent et al., 2019) do not recommend any currently available non-specific pharmacological treatment.

#### 4.1.1 Key findings of SC interventions for physical frailty in older adults

The allogeneic *h*BM-MSC preparation Lomecel-B (Longeveron, United States) is the leading SC preparation in the area of physical frailty. The CRATUS trial (Golpanian et al., 2017; Tompkins et al., 2017) showed that intravenous Lomecel-B was well tolerated and modestly but significantly increased the 6 MWD in frail elderly participants. The maximum increase in 6 MWD was obtained with the 100-million *h*BM-MSCs dose [+64 m in the phase II study of Tompkins et al. 2017)]. This 6 MWD value is higher than the “substantial meaningful change” (47–49 m) estimated by Perera et al. (2006).

The results of the CRATUS trial can also be compared with those obtained in a meta-analysis of 13 studies (Bohannon, 2007). In healthy subjects aged 70–79 years, Bohannon (2007) reported mean 6 MWD values of 510 m (490 m and 530 m for men and women, respectively). In the CRATUS trial (phase II sub-trial), the 100 million-*h*BM-MSCs-dose significantly increased mean 6 MWD from 345.9 m (baseline value) to 410.5 m (6-month value) (Tompkins et al., 2017). This means a recovery of about 39% of normal values for the elderly.

Another interesting observation from the CRATUS trial was a significant reduction in TNF-alpha levels in the groups treated with Lomecel-B (Golpanian et al., 2017; Tompkins et al., 2017). Acute inflammation is a natural defense process that eliminates infectious agents and toxins and promotes tissue repair (Rea et al., 2018; Furman et al., 2019). Aging and frailty are associated with chronic inflammation, driven by abnormal secretion of proinflammatory cytokines (at least in part by senescent cells). A review by Heinze-Milne et al. (2022) identified: 1) 31 of 37 studies reporting that circulating IL-6 levels increase with increasing degree of frailty, and 2) 9 of 17 studies reporting a positive association between TNF-alpha and frailty. In the CRATUS trial, serum TNF-alpha levels were significantly decreased in the Lomecel-B groups, but no significant changes in IL-6 were observed (Golpanian et al., 2017; Tompkins et al., 2017).

#### 4.1.2 Ongoing SC trials for physical frailty in older adults

##### 4.1.2.1 Ongoing trails with intravenous Lomecel-B

The positive results obtained in the CRATUS trial (Golpanian et al., 2017; Tompkins et al., 2017) were very encouraging to continue clinical development. Therefore, two additional RCTs (phase 2b NCT03169231 and phase 2 jRCT2043200038) were launched to further assess the efficacy and safety of Lomecel-B for aging frailty. The CRATUS trial also suggested that Lomecel-B might reduce age-related chronic inflammation (“inflammaging”). Therefore, a RCT (HERA, NCT02982915) was launched to evaluate the ability of Lomecel-B to improve influenza vaccine responses in frail subjects (Table 1).

Lomecel-B is also being evaluated in multiple clinical trials for aging-related conditions and chronic diseases (https://www.longeveron.com/clinical-pipeline). In 2021, the FDA granted Rare Pediatric Disease (RPD) designation for Lomecel-B (direct injection into the heart tissue) for the treatment of Hypoplastic Left Heart Syndrome, a rare and life-threatening congenital heart defect in infants (Longeveron, 2022b). Lomecel-B is undergoing clinical trial development for Alzheimer’s disease and Acute Respiratory Distress Syndrome (ARDS) due to COVID-19 (Longeveron, 2022a).

##### 4.1.2.2 hBM-MSCs derived from vertebrae from deceased donors (hvBM-MSCs)

An important limitation of BM-MSC therapy is the low number of cells obtained. This requires extensive cell expansion *ex vivo*, with the risk of cell senescence and reduced regenerative potency (Ganguly et al., 2017). Barbanti Brodano et al. (2013) compared the biological properties of MSCs derived from different sites in the human body, and found that *h*vBM-MSCs: 1) Can be maintained in culture for a greater number of passages, and 2) more efficiently generate mature cells of all mesenchymal lineages (osteogenic, adipogenic and chondrogenic differentiation). The number of *h*vBM-MSCs obtained from deceased donors is much higher than that obtained from traditional types of *h*BM-MSCs aspirated from living donors. NCT05284604 will assess the feasibility of *h*vBM-MSC therapy for aging frailty.

##### 4.1.2.3 hUC-MSCs

An *h*UC-MSC preparation investigated by the Shanghai East Hospital (China) recently completed a phase I/II study (Table 1). Moreover, a phase I/II RCTs (NCT04919135) and a phase I safety trial (NCT05018767) have been recently launched to evaluate the efficacy and safety of *h*UC-MSCs in aging frailty (Table 1).

*h*UC-MSC possess several advantages compared with *h*BM-MSCs: 1) The UC is a waste material, while the collection of *h*BM-MSC involves painful invasive procedures, and 2) the UC is a source of young SCs, while adult *h*BM-MSCs exhibit reduced cellular regenerative potency with increasing age (Ganguly et al., 2017). *h*UC-MSC have been extensively investigated to treat hematological disorders [reviewed in (Shang et al., 2021)], as well as to treat age-related or immune disorders [including metabolic and cardiovascular diseases and systemic lupus erythematosus; reviewed in (Xie et al., 2020)].

##### 4.1.2.4 hAD-MSCs

NCT03514537 is a safety trial of intravenous *h*AD-MSCs in frail adult and older adults (40–90 years) (Table 1). Comparisons with other studies are difficult to make because: 1) The doses of *h*AD-MSC used have not been found in the ClinicalTrials.gov database, 2) to my knowledge, no safety trial of intravenous *h*AD-MSC in frail subjects has been published previously, and 3) frail older people are susceptible to adverse drug reactions (Hilmer and Kirkpatrick, 2021).

##### 4.1.2.5 GMFFP

NCT03458429 investigates whether transfusion of GMFFP (GCSF-Mobilized Fresh Frozen Plasma) from young persons may be a safe and effective treatment for frailty and immune dysfunction in older people (Maharaj, 2020). A great advantage of GMFFP is that it is easy to collect and prepare in large quantities. However, there are some limitations regarding NCT03458429 (see Section 4.1.3).

#### 4.1.3 Limitations of current clinical trials

Current clinical trials with SCs for aging frailty use have three main limitations: 1) There is no standard protocol, 2) few pharmacokinetic and dosing data are available, and 3) SC interventions investigate older people, a “special population” who are poly-medicated and have comorbidities (Grimsrud et al., 2015).

##### 4.1.3.1 Lack of consensus on efficacy outcomes

The 6 MWD was one of the efficacy outcomes of the CRATUS trials with Lomecel-B (Golpanian et al., 2016; Golpanian et al., 2017; Tompkins et al., 2017), and 6 MWD is the primary outcome measure in the current multicenter trial of Lomecel-B (NCT03169231). Some but not all ongoing trials include 6 MWD as an outcome measure (see, for example, NCT04314011 in Section 3.1). These differences in evaluation tools do not allow for a precise comparison of efficacy results between clinical trials.

##### 4.1.3.2 Lack of consensus on diagnostic tools

A 6 MWD of >200 m and <400 m is an inclusion criterion of the current multicenter trial with Lomecel-B (NCT03169231), but this 6 MWD criterion was not included in the previous CRATUS trials (which included patients with CSF fragility scores of 4–7) (Golpanian et al., 2016; Golpanian et al., 2017; Tompkins et al., 2017). Differences in diagnostic tools are also common in ongoing trials (see Section 3.1) and make clinical efficacy comparisons even more difficult

##### 4.1.3.3 Very few pharmacokinetic studies of intravenous MSCs in humans are available

MSC distribution studies in rodents showed that 1) MSCs are transplantable by the intravenous route of administration, 2) more than 90% of intravenous MSCs are trapped in the lung and then cleared (by monocyte phagocytosis) with a half-life of 24 h and 3) local MSC administration is more appropriate for a regenerative effect *in situ* [for review, see (Elman et al., 2014; Salvadori et al., 2019)]. These results suggest that intravenous MSCs act, at least in part, through secreted factors.

Few studies have been dedicated to investigating the distribution of intravascular MSC in humans (Levy et al., 2020). In patients with myocardial infarction, Kang et al. (2006) showed that 2 h after intracoronary administration of radiolabeled H-MSCs, only 1.5% of the injected H-MSCs accumulated in the infarcted myocardium. Similarly, Hofmann et al. (2005) showed that after intravenous administration of radiolabeled BM cells, only background activity was detected in the infarcted myocardium. In men with localized prostate cancer, Schweizer et al. (2019) failed to detect intravenously infused allogeneic MSCs targeting the tumor.

##### 4.1.3.4 Further dosing studies are needed

Commenting on the CRATUS trial, Larrick and Mendelsohn (Larrick and Mendelsohn, 2017) noted that *“…modest improvement outcomes were limited to the lower dose, a finding that remains difficult to explain”,* and suggested that *“Future studies are definitely warranted given the magnitude of this increasingly important medical syndrome”.* Indeed, further studies in frail elderly are needed to optimize the dosage of intravenous MSCs, that is, to find the intravenous dose and frequency of administration that guarantee an optimal efficacy/safety ratio

##### 4.1.3.5 Influence of polypharmacy and comorbidities in frail elderly

Elderly subjects frequently have comorbidities, are poly-medicated, have reductions in hepatic and/or renal function, and have changes in the bioavailability of concomitant drugs (Grimsrud et al., 2015). In addition, such harm is amplified in frail people (Ibrahim et al., 2021).

In multiple myeloma patients older than 65 years and treated with autologous SC transplantation, Marini et al. (Marini et al., 2019) found that a reduction in the conditioning dose of melphalan was needed to maintain a safety profile similar to that in young subjects. Therefore, MSC therapy in a poly-medicated frail elderly patient may interact with concomitant medications, increasing or decreasing their bioavailability, with the risk of revealing adverse events, or reducing their therapeutic efficacy.

Older adults are considered by regulatory authorities to be a “special population” that has a therapeutic profile that cannot be directly extrapolated from what is known in adults (Grimsrud et al., 2015). Regarding safety issues, MSC therapies have a good safety profile, both in adults and in the elderly (Marini et al., 2019; Wang et al., 2021). However, severe comorbid disease is often considered an exclusion criterion in clinical trials, which carries the risk that the results obtained will be different from those obtained in “real life” conditions.

##### 4.1.3.6 Limitations concerning NCT03458429 with GMFFP

There are some limitations regarding NCT03458429 (GMFFP). First, file NCT03458429 (ClinicalTrials.gov database) assumes that GMFFP contains factors secreted by mobilized SCs, but does not specify which are present in the transfused preparations (Maharaj, 2020). Second, nothing was found in the medical literature to clarify this issue (a PubMed search using GMFFP as a keyword returned no results). Finally, the FDA issued a statement in 2019 (FDA, 2019) warning consumers not to receive plasma infusions from young donors that are promoted as an unproven treatment for various conditions, and NCT03458429 does not have a control (placebo) arm to assess the intervention efficacy.

##### 4.1.3.7 Negative results with NT-020

The nutrient combinations of NT-020 (NutraStem^®^) stimulate *h*SC proliferation *in vitro*, equal to or better than human granulocyte-macrophage colony-stimulating factor (*h*GM-CSF). However, NT-020 and exercise were unable to significantly increase blood levels of SCs in men and women aged 50–70 years, at two or 4 weeks from the start of the trial. Unlike the *in vitro* studies, *h*GM-CSF was not used as an active comparator

##### 4.1.3.8 Other SC preparations

No clinical trials for aging using E-SCs or induced pluripotent stem cells (iP-SCs) were found. The main reasons for this are: 1) their teratoma-forming tumorigenicity (Miyawaki et al., 2017), and 2) that the use of E-SCs raises ethical concerns (Lo and Parham, 2009).

#### 4.1.4 Future perspectives

The present investigation has not identified any SC preparation in late clinical development (Phase III RCTs) for physical frailty in older adults. Lomecel-B showed modest, but significant results in recent phase II RCTs (Golpanian et al., 2017; Tompkins et al., 2017), and if it successfully completes the current phase II RCTs, it would have the potential to initiate phase III trials and later become the first effective targeted intervention for aging frailty.

##### 4.1.4.1 What study protocol for phase III RCTs with SCs preparations?

**FIGURE 3:**
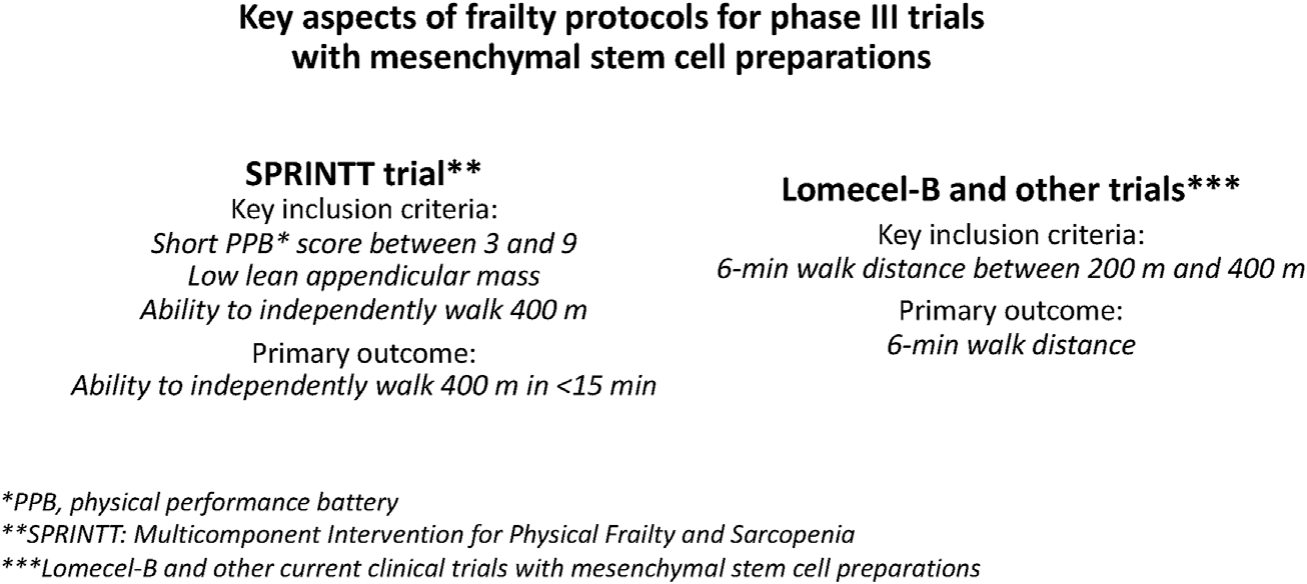
Key aspects of physical frailty protocols for phase III trials with mesenchymal stem cell (MSC) preparations. Two study protocols appear suitable for conducting phase III RCTs for aging frailty: (i) SPRINTT (Multicomponent Intervention for Physical Frailty and Sarcopenia) (Marzetti et al., 2018; Bernabei et al., 2022) and (ii) that used by ongoing clinical trials for aging frailty.

It would be preferable to perform phase III RCTs using a standard protocol, but this is not currently available. A previously proposed frailty protocol is the one used in the phase III RCT NCT02582138 (SPRINTT: Multicomponent Intervention for Physical Frailty and Sarcopenia) (Marzetti et al., 2018; Bernabei et al., 2022) (Figure 3). SPRINTT was designed to test interventions in older people with physical frailty and sarcopenia. The primary outcome is the ability to independently walk 400 m in <15 min. Inclusion criteria are: 1) short physical performance battery score between 3 and 9, 2) low lean appendicular mass, and 3) ability to independently walk 400 m (Marzetti et al., 2018; Bernabei et al., 2022). SPRINTT has the advantage of including the assessment of sarcopenia as an inclusion criterion.

In currently ongoing trials, 6 MWD is increasingly accepted as a primary outcome measure (and is also often an inclusion criterion, i.e., 6 MWD of >200 m and <400 m) (see Section 3.1). The 6MWT (6 Minute Walk Test) is simple, easy to perform, and better reflects activities of daily living than other walking tests (Chetta et al., 2006). Adopting it as the primary outcome to assess physical frailty (as well as inclusion criteria) may increase the relevance of comparisons with current clinical trials (Figure 3).

##### 4.1.4.2 Inflammatory biomarkers

In the CRATUS trial, serum TNF-alpha levels were significantly decreased in the Lomecel-B groups, but no significant changes in IL-6 were observed (Golpanian et al., 2017; Tompkins et al., 2017). Most of the clinical trials in Table 1 are investigating the effect of SC preparations on cytokine levels. Their results may help confirm the reduction in TNF-alpha levels as a valid criterion of efficacy (and/or better understand the role of IL-6 in therapeutic response).

##### 4.1.4.3 The efficacy of MSCs in reversing aging frailty can be improved

Most of the trials with MSC for aging frailty (Table 1) have not yet been completed (with results). If the results are not better than those obtained with Lomecel-B (Golpanian et al., 2017; Tompkins et al., 2017), other alternative types of SC preparations could enter preclinical and clinical research development.

###### 4.1.4.3.1 Preconditioning and/or genetic modification of naive MSCs

A large number of preclinical studies have shown that preconditioning naïve MSCs (with growth factors, drugs, and/or other factors), as well as genetic modification, can improve their therapeutic efficacy in many animal models of disease [for a review, see (Ocansey et al., 2020)]. The modified MSCs could enter cell therapy development, first in animal models of frailty [for animal models of frailty, see (Heinze-Milne et al., 2019; Heinze-Milne et al., 2022)] and then in clinical trials.

###### 4.1.4.3.2 Exosomes as another option to reverse the fragility of aging

Exosome therapies are intensively investigated in various clinical development programs. ClinicalTrials.gov lists 166 exosome intervention trials, but none for aging. The Chinese clinical trial database (chictr.org.cn/searchprojen.aspx) included a clinical trial with MSC-derived exosomes for photoaging (ChiCTR2200061216; see Section 3.2.3.2; Table 2). Interestingly, the exosomes used were loaded with circular RNA, a genetic modification procedure that can improve therapeutic efficacy [for information on genetic modification, preconditioning and engineering of MSC-derived exosomes, see (Ahmed and Al-Massri, 2022; Chen et al., 2022)].

Yoshida et al. (2019) reported that administration of nicotinamide phosphoribosyltransferase (NAMPT)-containing exosomes significantly enhanced wheel-running activity and prolonged lifespan in aged mice [for review of studies of MSC-derived exosomes in preclinical models of age-related diseases, see (Ahmadi and Rezaie, 2021); for perspectives and challenges of clinical trials with exosomes, see (Rezaie et al., 2022)]. Then, MSC-derived exosomes constitute an additional option to enter in preclinical and clinical development for the fragility of aging.

##### 4.1.4.4 Repurposing of approved SC products and related agents for aging frailty

Repurposing medicinal agents means finding new therapeutic indications for existing ones (Ayyar and Subramanian, 2022). Repurposing is a cost- and time-effective mechanism that can be applied to develop new SC therapies for aging frailty (Begley et al., 2021).

**FIGURE 4:**
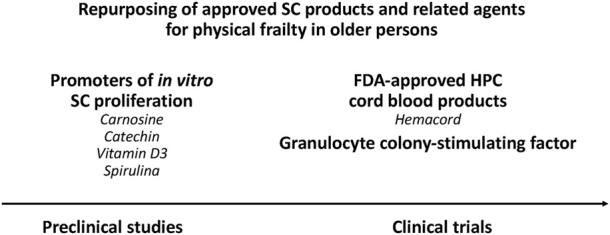
Repurposing of approved SC products and related agents for physical frailty in older persons. Several biological candidates can be repurposed for aging frailty: (i) Hemacord and other FDA-approved HPC cord blood products, (ii) the granulocyte colony-stimulating factor (G-CSF), and (iii) some nutraceuticals (carnosine, catechin, vitamin D3, spirulina).

Hemacord (BHI Therapeutic Sciences, United States) is an FDA-approved HPC cord blood product for disorders affecting the hematopoietic system (FDA, 2022). Hemacord is now in clinical development to be repurposed for acute ischemia stroke (NCT03735277), and could also be repurposed for aging frailty (Figure 4). It is important to mention that a large number of clinical studies are underway with allogeneic umbilical cord blood infusion for stroke and several other (non-hematopoietic) therapeutic indications (Ray and Mukherjee, 2021; Paton et al., 2022), but not for aging frailty. The current search identified only one study with allogeneic cord blood infusion for aging [case report of efficacy for a rare premature aging disorder, the Hutchinson-Gilford progeria syndrome (Suh et al., 2021)].

Darvadstrocel (Alofisel^®^, Takeda Ireland) and holoclar (Holoclar^®^, Holostem Terapie Avanzate, Italy) are EMA-approved SC products, but neither manufacturer has announced the launch of clinical trials for aging conditions (https://www.takeda.com/worldwide/) (https://www.holostem.com/news/?lang=en).

An important candidate to be repurposed for aging frailty is G-CSF (Figure 4). The G-CSF receptor (G-CSFR) is expressed in mouse and human skeletal muscle (Hara et al., 2011; Wright et al., 2014). Rodent studies have shown that: 1) Following skeletal muscle injury, G-CSF administration enhances satellite cell proliferation and muscle strength (Stratos et al., 2007), and 2) G-CSF enhances load-induced muscle hypertrophy (Ohashi et al., 2018). More importantly, muscle secreted G-CSF ameliorated satellite cell loss in the muscle of aged mice (Li et al., 2019).

Some nutraceuticals (carnosine, catechin, vitamin D3, spirulina) promote SC proliferation *in vitro* (Bickford et al., 2006; Bachstetter et al., 2010), and are candidates to be repurposed for aging frailty (Figure 4). Before entering clinical trials for aging frailty, these compounds should show their ability to increase SC blood levels in animal models (using GM-CSF as active comparator).

##### 4.1.4.5 MSCs as “longevity candidates”

Aging frailty is associated with increased all-cause mortality (Grabovac et al., 2019) suggesting that Lomecel-B and other MSC therapies may increase life expectancy in frail older persons [for the relationship between longevity and all cause-mortality, see (Garay, 2021)]. In addition, animal studies consistently showed increased life expectancy with MSC transplantation (Shen et al., 2011; Lavasani et al., 2012; Mansilla et al., 2016). Therefore, MSCs can be considered as “longevity candidates”.

The National Institute on Aging (NIA, United States) has launched an Intervention Testing Program (ITP) dedicated to identifying longevity drug candidates in mice (Nadon et al., 2017), for further testing in human clinical trials (Garay, 2021) (https://www.nia.nih.gov/research/dab/interventions-testing-program-itp). Therefore, MSCs deserve to be investigated for their ability to increase healthy lifespan in the ITP.

### 4.2 Facial skin aging

Various non-invasive methods have been used to prevent or treat facial aging, such as creams and lotions, without really satisfying results. In contrast, autologous fat grafting is efficacious for facial plastic and reconstructive purposes, and is widely used to restore volume and improve skin quality (facial skin rejuvenation) (Vasavada and Raggio, 2022).

#### 4.2.1 Key findings of SC interventions for facial skin aging

Facial skin rejuvenation is another area of clinical research with SC preparations [for a review, see (Surowiecka and Struzyna, 2022)]. Recently, an RCT by Yin et al. (2020) clearly showed that SVF-assisted autologous fat grafting increases graft survival, facial volume, and skin quality.

#### 4.2.2 Ongoing clinical trials for facial skin aging

##### 4.2.2.1 hSVF preparations for facial skin aging

Two RCTs (NCT03928444 and RPCEC00000362) and one single-arm clinical trial (IRCT20141007019432N2) are investigating *h*SVF preparations for facial rejuvenation (Table 2). The positive results obtained by Yin et al. (2020) with autologous *h*SVF transplantation suggest that similar protocols could be used in other clinical settings for regulatory purposes (see Section 4.2.3)

##### 4.2.2.2 AD-MSCs “secretome” preparation

The SC “secretome” comprises diverse soluble factors (chemokines, cytokines, growth factors, angiogenic factors, and exosomes) produced in the endosomal compartment, and released for SC migration, apoptosis, proliferation, and angiogenesis [for review, see (Xia et al., 2019)]. Recent work suggests that the regenerative mechanism of SC transplantation could involve a modulatory paracrine effect of the SC secretome (Xia et al., 2019).

Compared to SC preparations, SC secretome has several advantages, including ease of manufacture, freeze-drying, packaging, and easier transportation (Xia et al., 2019). In addition, the SC secretome has shown potential to counteract facial aging (Kerscher et al., 2022). PT Kimia Farma Tbk (Jakarta, Indonesia; https://www.kimiafarma.co.id/) develops an *h*D-MSC secretome preparation for facial aging. This preparation is being investigated in NCT05508191, which compares two methods of secretome administration (microneedle and fractional CO₂ laser).

#### 4.2.3 Weakness of clinical research with hAD-MSCs for facial skin aging

##### 4.2.3.1 SVFs are regulated as biologicals in the US

A large number of studies with SVFc for facial rejuvenation have been conducted in the US (Surowiecka and Struzyna, 2022), but very few are registered on ClinicalTrials.gov (Table 2). One important reason is that the FDA regulates SVF preparations as biologics, because mechanical processing is required (ASCS, 2019). This means that any surgeon wishing to use SVF preparations must submit an Investigational New Drug Application (INDA) to the FDA and be approved by an ethics committee. Consequently, the FDA initiated legal action against SC clinics using unauthorized SC products (El-Kadiry et al., 2021).

##### 4.2.3.2 RCT in surgery are difficult to conduct

The efficacy results of the available SC studies are difficult to compare due to the very different techniques used for the extraction of fatty tissue, and for preparation and injection of SVFs (Surowiecka and Struzyna, 2022). In addition, outcome results are not similar for all surgeons (Demange and Fregni, 2011). Robinson et al. (2021) analyzed 388 RCT in surgery and identified several limitations: 1) trial registration was suboptimal, 2) sample sizes were small, 3) only a few trials were focused on major clinical events, and 4) few trials controlled the quality of the intervention or the experience of the surgeon.

#### 4.2.4 Future perspectives

##### 4.2.4.1 Research advantages of clinical trials on facial aging

Regardless of aesthetic considerations, clinical trials with SC preparations for facial rejuvenation are important because: 1) Tissue regeneration is directly assessed by sophisticated and precise methods and 2) SC trapping in the lung (Elman et al., 2014; Salvadori et al., 2019) and safety problems of intravenous MSC administration are avoided

##### 4.2.4.2 Regulatory aspects of clinical trials with autologous SVFs preparations

The FDA regulates allogeneic SVF products as biologics (ASCS, 2019), but the risk of skin rejection prevents clinical development of allogeneic SVF preparations for facial rejuvenation. The final consequence is that the very numerous US clinical trials with autologous SVF preparations for facial rejuvenation (Surowiecka and Struzyna, 2022) are not registered on ClinicalTrials.gov.

Some RCT with autologous SVF preparations have recently been registered on ClinicalTrials.gov (see Table 2). The application of similar RCT protocols could pave the way for autologous SVF preparations to be registered on ClinicalTrials.gov and comply with FDA regulations (Investigational New Drug applications and FDA-approvals).

#### 4.2.5 Cutaneous photoaging

Photoaging of the skin (“EJ20 Photoaging of the skin”) was recognized as a disease in the WHO ICD-11 (https://icd.who.int/browse11/l-m/en#/http://id.who.int/icd/entity/673647195). Topical retinoids such as tretinoin are effective in improving the clinical appearance of sun-damaged skin (Serri and Iorizzo, 2008). Topical treatment is often supplemented with orally administered vitamins, polyphenols, and carotenoids (Parrado et al., 2018). Among surgical approaches, fractionated laser is widely used for treating cutaneous signs of photoaging (Wu et al., 2016).

Preclinical studies suggested that AD-MSC preparations possess anti-wrinkling properties (Chen et al., 2020), and Charles-de-Sa et al. (Charles-de-Sa et al., 2020) recently reported full regeneration of solar elastosis by subdermal AD-MSC injection.

There are two ongoing clinical trials for cutaneous photoaging: 1) NCT01771679 which was evaluating intravenous allogeneic *h*BM-MSCs for the treatment of facial photoaging (recruitment was suspended), and 2) ChiCTR2200061216 which is currently exploring whether *h*ADSC-derived exosomes (loaded with circcol elns) can promote collagen and elastin synthesis in photoaged skin (Table 2).

Circular RNAs (circRNAs) include a large family of non-coding RNAs, which can regulate gene expression (acting on transcription, mRNA turnover and translation, by sponging microRNAs and RNA-binding proteins) (Panda, 2018). Intensive research of non-coding RNA therapy for photoaging is currently being carried out at Sun Yat-Sen University Hospital (China) (ChiCTR2200061216, Table 2) (Peng et al., 2017; Hou et al., 2021). If ChiCTR2200061216 yields positive results, treatment with circcol elns-loaded exosomes would hold great promise for cutaneous photoaging.

## 5 Concluding remarks

Clinical research with SC therapies for aging focuses on two main objectives: Physical frailty and facial skin aging. The advantages and disadvantages of these two objectives are complementary (which facilitates a global vision). Physical fragility affects organs that are usually accessed parenterally, where the pulmonary filter makes it even more difficult for SCs to access the target organ. Rejuvenating the skin is above all an aesthetic objective, but the effectiveness is evaluated directly on a visible and easily accessible organ.

With regard to aging frailty, the allogeneic *h*BM-MSC preparation Lomecel-B (Longeveron, United States) is the leading SC preparation in the area (Golpanian et al., 2016; Golpanian et al., 2017; Tompkins et al., 2017; Longeveron, 2021a; Longeveron, 2021b; Yousefi et al., 2022). Positive results have been obtained in preliminary phase II studies. An *h*UC-MSC preparation investigated by the Shanghai East Hospital (China) recently completed a phase I/II study. Several other clinical trials are currently underway for aging frailty (Table 1).

Facial skin aging is another area of clinical research with SC preparations. An RCT conducted by Yin et al. (2020) has shown positive results with an autologous *h*SVF preparation. Several other clinical trials are currently underway for facial skin aging (Table 2).

Clinical research with SC interventions for aging has only recently begun. This area of research has received a great initial impetus, as demonstrated by the twenty clinical trials launched worldwide and reviewed here. Let’s hope that all these efforts will be rewarded with the arrival of the first SC anti-aging product in the near future.
